# Acute upper and lower gastrointestinal bleeding management in older people taking or not taking anticoagulants: a literature review

**DOI:** 10.3389/fmed.2024.1399429

**Published:** 2024-05-03

**Authors:** Danilo Menichelli, Gianluca Gazzaniga, Francesco Del Sole, Arianna Pani, Pasquale Pignatelli, Daniele Pastori

**Affiliations:** ^1^Department of Clinical Internal, Anesthesiological and Cardiovascular Sciences, Sapienza University of Rome, Rome, Italy; ^2^Department of General Surgery and Surgical Specialty Paride Stefanini, Sapienza University of Rome, Rome, Italy; ^3^Department of Medical Biotechnology and Translational Medicine, Postgraduate School of Clinical Pharmacology and Toxicology, Università degli Studi di Milano, Milan, Italy; ^4^Department of Oncology and Hemato-Oncology, Università degli Studi di Milano, Milan, Italy

**Keywords:** endoscopy, older population, anticoagulants, gastrointestinal bleeding, proton pump inhibitors

## Abstract

Acute upper and lower gastrointestinal (GI) bleeding may be a potentially life-threatening event that requires prompt recognition and an early effective management, being responsible for a considerable number of hospital admissions. Methods. We perform a clinical review to summarize the recent international guidelines, helping the physician in clinical practice. Older people are a vulnerable subgroup of patients more prone to developing GI bleeding because of several comorbidities and polypharmacy, especially related to an increased use of antiplatelet and anticoagulant drugs. In addition, older patients may have higher peri-procedural risk that should be evaluated. The recent introduction of reversal strategies may help the management of GI bleeding in this subgroup of patients. In this review, we aimed to (1) summarize the epidemiology and risk factors for upper and lower GI bleeding, (2) describe treatment options with a focus on pharmacodynamics and pharmacokinetics of different proton pump inhibitors, and (3) provide an overview of the clinical management with flowcharts for risk stratification and treatment. In conclusion, GI is common in older patients and an early effective management may be helpful in the reduction of several complications.

## Introduction

Gastrointestinal (GI) bleeding is one of the most frequent gastroenterological conditions that require medical attention. Its incidence varies with age, with older patients being more frequently affected. The severity of gastrointestinal bleeding can vary from a mild form to a potentially threatening life condition. The estimated prevalence for overall GI bleeding is approximately 3.1% (1). The origin of the bleeding defines its clinical presentation and definition, with hemorrhages originating before the Treitz ligament being classified as upper GI bleeding and those originating after this landmark as lower GI bleeding.

Upper GI bleeding in the older population is frequently associated with gastric and duodenal ulcer or esophagitis, being responsible for the episode in 80% of the cases (2). The incidence of upper GI bleeding can also vary depending on the geographical region. In Northern Europe, the annual incidence ranges from 213 to 570 per 100,000 patients (3); in a UK cohort of older patients affected with acute upper GI bleeding, the rate was 63% in those above 60 years of age (4); and in a North American cohort, this rate ranged between 35 and 45% (5). Although the incidence of upper GI bleeding in older patients is high, there is an even higher incidence of lower GI bleeding (6).

The incidence of hospitalizations for upper GI bleeding increased with age, being 197.4 per 100,000 population between 66 and 75 years of age and rising to 425.2 per 100,000 in people older than 75 years in a North American Cohort (7). In addition, the risk of readmittance after a first hospitalization remains higher than that of the general population.

As the risk of complications increases, so does the risk of mortality in a patient aged over 60 years experiencing an upper GI bleeding episode, estimated to be between 12 and 25%, compared to the 10% of the general population (2). In a study based on the Welsh population, the case fatality for an upper GI bleeding ranged from 11.2 to 21.5% in men above 65 years of age and from 9.1 to 20.7% in women, increasing with age (8).

Lower GI bleeding is a condition that most frequently appears in older patients, with an incidence that also depends on geographic and socio-economic factors and comorbidities (3). The most common etiologies are diverticulosis, ischemic colitis, colitis, hemorrhoids, and colorectal cancer (3). In Northern Europe, the incidence rate for lower GI bleeding ranges from 2.41 to 3.64 in male patients and from 1.72 to 3.10 in female patients, increasing with age (9). In a Spanish study, the incidence of new lower GI bleeding was between 100 and 150 per 100,000 patients in 2005 (6).

With the higher frequency of comorbidities, this cohort of patients also has an increased risk of hospitalization and longer in-hospital stay (10), with an estimated rate between 127.7 and 380.1 per 100,000 population, increasing with age (7). A multicentric study in a European cohort shows that this cohort of patients also suffers from an increased mortality rate, where the hospital mortality for lower GI bleeding was 2.5 and 1.17% in the following 3 months (1).

## Pharmacological history

Medication history should be carefully reviewed upon admission, as many drugs may be associated with gastrointestinal bleeding.

First, anticoagulants and antiplatelets increase bleeding risk (11, 12). Their prescription is very common, as it has been reported that about half of the patients presenting for an upper gastrointestinal bleeding (UGIB) were treated using antithrombotic drugs (13). Clinicians should assess the risk–benefit ratio of antithrombotic administration in this setting. Indeed, as shown by the ASPREE trial enrolling 19,114 healthy older people (>65 years) patients without previous cardiovascular disease, the administration of low-dose aspirin increased the risk of major bleeding (hazard ratio [HR] 1.38, 95% confidence interval [95%CI] 1.18–1.62) and UGIB (HR 1.87, 95%CI 1.32–2.66) without an improvement of cardiovascular disease prevention (14). Furthermore, recent European guidelines advise that the use of aspirin for primary cardiovascular prophylaxis should be discontinued in patients who have a confirmed UGIB, although it should be continued for secondary prevention (15). Similarly, people taking warfarin should have the medication stopped along with anticoagulant reversal in situations of severe UGIB, while continued anticoagulation must be evaluated in cases of less severe UGIB (15).

Second, non-steroidal anti-inflammatory drugs (NSAIDs) are commonly prescribed in adult people; they represent a common risk factor for gastric hemorrhage. Their primary mechanism is the inhibition of cyclooxygenase 1 (COX-1); as a consequence, there is a great reduction of prostaglandin production, which leads to poorer protection of gastric mucosa. Coadministration of aspirin and other NSAIDs have been associated with greater gastric damage because of combined inhibition of both COX-1 and COX-2 mucosae-protective pathways (16).

Beyond these medications, other drugs have been associated with a potentially increased risk of GI bleeding, such as serotonin-selective reuptake inhibitors and calcium channel blockers; therefore, patients should be questioned whether they have been prescribed these drugs as well (17, 18).

Moreover, drugs *per se* may induce upper bleeding by causing pill esophagitis. A large number of medications have been associated with this phenomenon, with antibiotics, NSAIDS, and bisphosphonates being the most common (19). This occurrence is more frequent in older adults with a reduced esophageal transit; therefore, it should always be excluded in case of retrosternal pain, odynophagia, and drug assumption before sleeping up to 3 days before and whether the real cause of bleeding has not been identified yet (19).

Finally, the patient should be asked about recent consumption of products that may change stool appearance and make it look similar to real bleeding (i.e., iron, bismuth, liquorice, and charcoal), in order to avoid a wrong diagnosis in the initial stages (20, 21).

## Pharmacological treatment

### Anti-acid treatment

Pharmacological suppression of gastric acid production is routinely performed in patients with UGIB upon admission. This effective strategy is based on the reason that the coagulation process benefits from a higher than normal gastric pH, which, therefore, leads to a better control of hemorrhage (22).

However, despite this physio-pathological mechanism, not all acid-suppressive strategies have proved to be equal; a meta-analysis of 11 studies highlighted that H_2_ receptor antagonists are less efficient at stopping recurrent or persistent hemorrhage than proton pump inhibitors (PPIs), in particular for Forrest Ia, Ib, or IIa ulcers (23). For this reason, although there is evidence for a potential role of this drug class in ulcer prevention (24), it is not routinely prescribed in this setting.

There is no clear evidence on the best schedule of PPI use in terms of time, type of administration, and type of PPI. Intravenous (IV) formulation is usually preferred; however, if not available, the oral formulation may be used as well, as it has been suggested to have a similar effectiveness (25).

A recent Cochrane meta-analysis of RCTs has investigated the pre-endoscopic PPI role. There are insufficient data to determine high-certainty evidence; however, it seems that PPI administered before endoscopic procedures may not reduce mortality and need for surgery (26). On the contrary, they may reduce rebleeding and the need for hemostatic treatment performed at index endoscopic procedures with low and moderate certainty of evidence, respectively (26).

Several PPIs are available and widely used (Table 1); however, there are no recommendations on the best one to administer in this setting. As reported, they have similar pharmacokinetic parameters such as high bioavailability, volume of distribution, and protein binding. However, some differences may induce a preference according to specific situations.

**Table 1:** Comparison of proton pump inhibitors: pharmacokinetics and special populations (27, 28).

		Omeprazole	Esomeprazole	Pantoprazole	Lansoprazole	Rabeprazole
Pharmacokinetic parameters	Formulations	Oral, IV	Oral, IV	Oral, IV	Oral	Oral
	Absorption (Tmax)	0.5–3.5 h	1.5 h	2–3 h	1.7 h	1 h
	Bioavailability	30–40%	64–90%	77%	80–90%	52%
	Half-life	0.5–1 h	1–1.5 h	1 h	0.9–2.1 h	1–2 h
	Volume of distribution	0.3 L/kg	16 L	11.0–23.6 L	0.4 L/kg	NA
	Protein Binding	95%	97%	98%	97%	96.3%
	Metabolism	Mainly CYP2C19, then CYP3A4	Mainly CYP2C19, then CYP3A4	Mainly CYP2C19, then CYP3A4	Mainly CYP3A4 then CYP2C19	Mainly CYP2C19, then CYP3A4
	Elimination	Renal (77%), biliary	Renal (80%), biliary	Renal (80%), biliary	Renal (14–23%)	Renal (90%)
	Dietary considerations	0.5–1 h before meal	1 h before meals (otherwise only 43% bioavailability) not clinical significative levels	Zinc supplementation may be needed in zinc-deficient patients because of IV formulation with EDTA	1 h before meals Phenylalanine may be contained in certain formulations.	Capsules: 0.5 h before meal (Exception: tablets for duodenal ulcers or *Helicobacter pylori* eradication administered after/with meal, respectively)
Special populations	Renal failure	No dosage adjustment necessary	No dosage adjustment necessary	No dosage adjustment necessary	No dosage adjustment necessary	No dosage adjustment necessary
	Hepatic impairment	Dosing A maximum dose of 20 mg/day, regardless of indication has been proposed PK Bioavailability and plasma half-life are increased; plasma clearance is decreased.	Dosing Child-Pugh A-B: No dosage adjustment necessary. Child-Pugh C: variable according to indication (20 mg OD–BID)	Dosing No dosage adjustment necessary; doses >40 mg daily have not been evaluated. PK: Increase in serum elimination half-life; AUC increases by 5-7x.	Dosing Child-Pugh A-B: No dosage adjustment necessary. Child-Pugh C: 15 mg once daily. (Based on 30 mg OD schedule) PK in mild and moderate hepatic impairment, AUC, and half-life increased ~3x.	Dosing Child-Pugh A-B: No dosage adjustment necessary. Child-Pugh C: Avoid use; if necessary, monitor cautiously for adverse reactions. PK, AUC and half-life approximately 2x, total clearance decreased to less than half,
	Older people	Bioavailability may be increased, while elimination rate is decreased	AUC and C_max_ were increased by 25 and 18%, respectively	Moderate increase in Cmax (26%) and AUC (43%) after oral administration	Clearance is decreased with t½ increasing ~50 to 100% ➔ mean t½ = 1.9–2.9 h ➔ repeated OD dosing does not accumulate	AUC values approximately 2x; Cmax increased

AUC, area under the curve; EDTA, ethylenediaminetetraacetic acid; GERD, gastroesophageal reflux disease; IV, intravenous; OD, once daily; PK, pharmacokinetics.

Half-lives range from 0.5 to 3 h; given this short period and the fact that not all protonic pump inhibitors are targetable at the same time, a three-day period has been estimated to reach a steady-state inhibition of gastric acid (29). Despite the slightly different half-lives of PPIs, the drug of choice should not be determined on this feature as they all cause irreversible and durable inhibition of protonic pumps. Considering that preclinical models have shown that H^+^, K^+^-ATPase has a half-life of approximately 54 h (29), it must be considered that the effects of PPIs do not cease right after the last dose administration.

All the PPIs undergo hepatic metabolism; in particular, CYP2C19 plays a relevant role, followed by CYP3A4. This aspect should be taken into account as CYP2C19 genetic polymorphisms may give an extensive or poor-metabolizer phenotype, which reflects in a lower or higher PPI exposure, respectively (29); in case of genetic variants, esomeprazole should be preferred to omeprazole as its metabolism is less influenced by CYP2C19 polymorphisms (30).

PPIs are eliminated by the kidney; this is less true for lansoprazole, as the kidney accounts only for 14–23% of its elimination. In any case, no dosage adjustment for kidney function is required, regardless of PPI type and severity of the renal disease.

PPI treatment is usually chronic after the acute bleeding episode; older people commonly have concomitant diseases, which require other treatments as well. It has been estimated that 55–98% of people over 65 years old have at least two comorbidities (31). A higher disease burden implies a higher number of medications prescribed, thus leading to a higher risk of interactions among drugs. PPIs may interact with other medications in several ways.

First, PPI-induced modulation of gastric pH may result in reduced bioavailability of certain drugs administered orally; as an example, it has been reported that coadministration of omeprazole may lower bioavailability of methotrexate, ketoconazole, mycophenolate mofetil, and protease inhibitors by affecting their solubility; for this reason, their pharmacokinetic profile may be altered (32).

Second, a certain potential of PPIs to interact with intestinal P-glycoprotein (P-gp) cannot be excluded; this may be an issue, as many P-gp substrates, such as digoxin, nifedipine, amitriptyline, and tacrolimus, are widely administered in people over 65 years old; for this reason, coadministration must be carefully monitored (33).

As previously stated, PPIs are mainly metabolized by CYP2C19; this may lead to drug–drug interactions (DDI) with pharmacological agents that are substrates of the same enzymes. Among the PPIs, omeprazole has a higher DDI potential given its strong affinity for CYP2C19 and CYP3A4; it has been reported to have interaction with diazepam, moclobemide, phenytoin, and warfarin (32). In case of concomitant drugs, which may have an interaction, there is some evidence that pantoprazole, rabeprazole, and lansoprazole may carry a weaker risk of interactions (32).

Interaction between omeprazole/esomeprazole and clopidogrel is of high clinical relevance in this setting and should be monitored: It has been demonstrated that concomitant administration of omeprazole is associated with a lower exposure to active clopidogrel metabolite, regardless of a possible double dose of clopidogrel or a 12-h time period between the administrations of the two drugs (32, 34). In this case, pantoprazole should be preferred given its lower influence on clopidogrel metabolism.

Beyond pharmacological interactions, numerous studies have shown that PPIs are linked to an increased risk of a variety of negative effects, including *Clostridium difficile* infection, osteoporotic-related fractures, renal impairment, community-acquired pneumonia, vitamin B12 deficiency, and dementia (35). Given the range of potential side effects linked to long-term use in older people, an assessment for the need to continue PPI therapy should be routinely conducted.

This is the reason why guidelines for PPI deprescribing have been developed. A periodic assessment of PPI indications should be conducted regularly, to lower exposure; however, discontinuation is not indicated for patients with severe gastro-esophageal diseases (36).

### Vasoactive drugs

Pharmacological agents acting on vasoconstriction (e.g., terlipressin, octreotide, and somatostatin) are recommended in addition to endoscopy in patients with UGIB from varices (or at risk for varices) (37); therapy should last from presentation to 3–5 days after bleeding cessation.

As a class, they are associated with a better hemostasis, lower need of blood transfusions, and a lower risk of 1-week mortality (38).

Among the others, terlipressin (2 mg IV q4h and then 1 mg IV q4h) has proved a 34% relative risk reduction in mortality (39). From a pharmacological point of view, terlipressin is a vasopressin analog, which acts by constricting mesenteric artery; this leads to a lower portal venous flow and, therefore, to a lower portal pressure.

Octreotide is a synthetic analog of somatostatin, a hormone which reduces release of vasodilators, thus causing a reduced portal inflow. It is administered by bolus 50 mcg IV, followed by continuous infusion (CI) 50 mcg IV each hour, and is the most widespread choice in USA in these cases, as terlipressin is not available in this country. Compared to somatostatin, octreotide has a longer half-life, but causes a similar prompt reduction of variceal pressure; however, despite adding continuous infusion, these effects only last some minutes, probably due to a pharmacodynamic desensitization (40). Nevertheless, a longer term effect mediated by other pathways may not be excluded (41).

When compared with octreotide, in a randomized controlled trial (RCT) of cirrhotic patients, terlipressin has shown a longer effect in reducing portal pressure (42); therefore, it should be preferred if available.

No vasoactive treatments should be used in place of endoscopic variceal ligation.

### Prokinetics

Prokinetic drugs may be administered as they help in cleaning the stomach from blood clots and other residues, thus allowing endoscopist have a better visualization of active bleeding sources.

Erythromycin has been studied in this setting due to its role as motilin receptor agonists. A meta-analysis of RCT has proved that it may improve visualization of gastric mucosa (43), while another showed that it is statistically associated with a lower rate of second-look endoscopies and a shorter length of stay in hospital (44). There is no clear evidence on whether adding erythromycin has a further benefit compared to nasogastric tube lavage only (43).

Notably, erythromycin is a strong CYP3A4 inhibitor: In older patients, this may represent an issue as concomitant drugs are often administered and their metabolism may be altered (45). Similarly, it has been associated with QTc prolongation and a higher risk of torsades de pointes, which should be taken into account in this population (46). Obviously, the shorter the exposure to this drug, the lower the risk of clinically significant DDI, which, however, may not be excluded.

It may be argued that, given the similar mechanism, metoclopramide may have a role as well. However, previous evidence already discussed metoclopramide role and found no effect. For this reason, despite a similar function, erythromycin is currently preferred to metoclopramide in UGIB (47).

### Antibacterial treatment during GI bleeding

Patients with cirrhosis and GI bleeding are frequently diagnosed with bacterial infections; approximately 22% of patients develop an infection in the first 2 days of hospital stay, while this incidence peaks up to 66% considering the first 2 weeks (48). For this reason, an antibiotic prophylaxis is usually administered in cirrhotic patients with gastrointestinal hemorrhage. A large spectrum antibiotic prophylaxis has been associated with a lower mortality, rate of bacterial infections, rebleeding rate, and length of hospitalization (49). Broad-spectrum antibiotics should be started before endoscopy and administered for up to 7 days (37).

A usual choice may be ceftriaxone 1 g once daily (OD) IV; If the patient is discharged before a week, change to ciprofloxacin 500 mg bis in die (BID) may be an alternative, although ceftriaxone has proven to be superior (37). However, drug should be selected considering patients characteristics, such as comorbidities and previous exposure to antibiotics, as people above the age of 65 years may have hepatic or renal impairments, which may affect drug metabolism and elimination; for instance, a pharmacokinetic study on older patients with moderate-to-severe impairment in renal function has highlighted a greater ceftriaxone exposure with a 48 h dosing schedule (50). Similarly, bacterial characteristics should be considered as well, as a local pattern of ceftriaxone and quinolone resistance in cirrhotic patients have been reported (51, 52).

However, despite antibiotic administration, bacterial infections still occur in approximately one-fifth of cirrhotic patients admitted for variceal bleeding; therefore, it still remains a crucial issue that should be carefully taken into account (53).

## Management of acute upper GI bleeding

Upper gastrointestinal bleeding (UGIB) is defined as hemorrhage proximal to the Treitz ligament involving the esophagus, stomach, and duodenum (54). The most common symptoms of UGIB are melena, hematemesis, and coffee ground vomiting (54). Hematochezia, instead, is a rare manifestation of UGIB and is commonly a presentation of lower gastrointestinal bleeding (LGIB) (55, 56). Systemic manifestations, in major and life-threatening gastrointestinal (GI) bleeding (both UGIB and LGIB), include hemodynamic instability, hypotension, abdominal pain associated with lethargy, fatigue, syncope, and angina (56, 57).

The most common cause of UGIB is peptic ulcer disease, involving approximately 32–36% of all hospitalized patients; then esophagitis, duodenitis, and gastritis (until 24% of hospitalization); and finally variceal bleeding (approximately 11% of hospitalization, but 90% of UGIB in patients with liver cirrhosis) (4, 56, 58, 59).

The incidence of UGIB is widely different among countries ranging between 67 and 172/100.00 person with similar rates between Europe and the United States (59–61). Although hospitalizations for UGIB have declined due to *H. pylori* eradication, the use of proton pump inhibitors, and increased access to endoscopy (59, 62, 63), the mortality rate of UGIB is approximately 2–10% (54).

Antiplatelet and anticoagulant use, non-steroidal inflammatory drugs, corticosteroids, liver cirrhosis, the presence of multiple comorbidities, and older age are common risk factors for gastrointestinal bleeding, especially of UGIB (1, 6, 56, 62).

**Figure 1:**
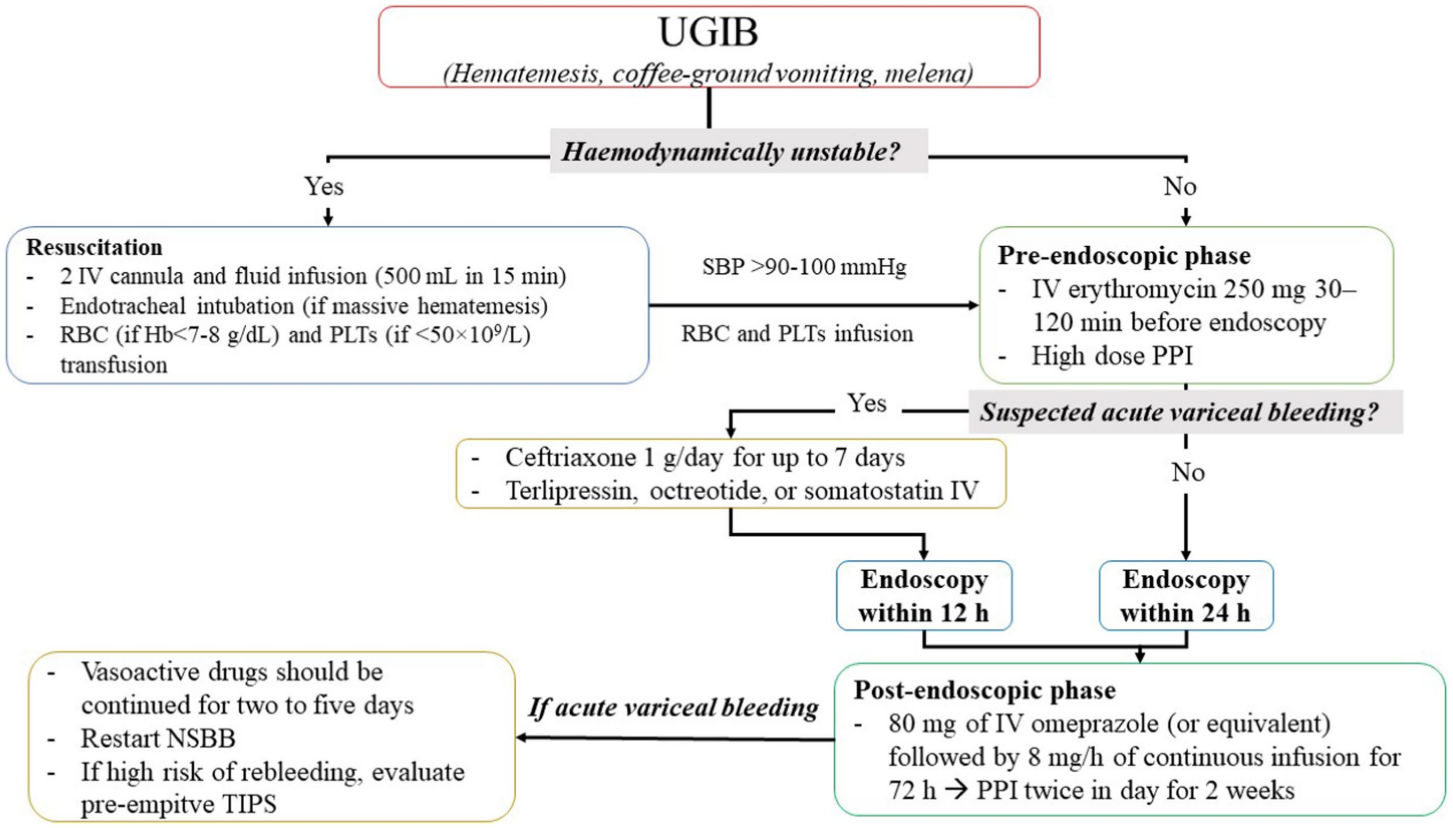
Flowchart of upper gastrointestinal bleeding management. IV, intravenous; NSBB, non-selective beta-blockers; PLTs, platelets; PPI, proton pump inhibitors; RBC, red blood cells; SBC, systolic blood pressure; TIPS, transhepatic intrajugular portosystemic shunt; UGIB, upper gastrointestinal bleeding.

Several guidelines suggest general recommendations for initial management of UGIB (a flowchart of UGIB management is proposed in Figure 1). First, patients with UGIB should be guaranteed an IV access by cannula (≤18 G) in each antecubital fossa and an early fluid resuscitation (37) should be started, reducing the risk of mortality and myocardial infarction (54), achieving 90–100 mmHg systolic blood pressure as target (64, 65). In particular, a first approach with 500 mL of crystalloids infused in less than 15 min are suggested as first choice in hemodynamically unstable patients (66), although studies showed no difference between colloids and crystalloids in fluid resuscitation during UGIB (54).

During resuscitation, transfusion of packed red cells should be performed with a restrictive approach using a threshold of 7 g/dL (<8 g/dL in patients with cardiovascular disease) for hemoglobin (Hb) (37, 54) and transfusion of platelets should be performed using a threshold of 50 × 10^9^/L (67, 68). After resuscitation, treatment of UGIB is divided into three phases: pre-endoscopic, endoscopic, and post-endoscopic phases.

### Pre-endoscopic phase

#### General recommendations

In this phase (Figure 1), if patients are hemodynamically stable, erythromycin (250 mg IV infusion approximately 30–120 min before endoscopic procedures) was administered: Indeed, erythromycin, as a prokinetic agent, improves visualization during the endoscopy procedure resulting in a lower length of hospital stay, a lower rate of re-intervention, and less need for blood transfusions (37, 54). Furthermore, a large meta-analysis conducted by the Cochrane Institute showed that PPI may be useful (26) and performed on 2,223 patients included in six RCTs. Indeed, there is moderate-certainty evidence that PPI started before endoscopy for UGIB likely reduces the requirement for endoscopic hemostatic treatment. However, there is insufficient evidence to conclude whether PPI had a role on mortality, rebleeding, and need for surgery.

On the other hand, all guidelines recommended against the use of tranexamic acid in UGIB due to high risk of venous thromboembolism without an improvement on mortality (37, 54, 69).

#### Variceal bleeding

Patients with compensated advanced chronic liver disease and clinically significant portal hypertension defined as hepatic venous pressure gradient>10 mmHg and/or liver stiffness by transient elastography >25 kPa should be treated for nonselective beta blocker (NSBB) as carvedilol to prevent the development of variceal bleeding (37). For patients unsuitable for NSBB and with high-risk esophageal varices, endoscopic band ligation is the endoscopic prophylactic treatment of choice (37). Of note, in patients with advanced chronic liver disease and portal vein thrombosis, an anticoagulation treatment, if not contraindicated, may be helpful to prevent variceal bleeding: indeed, in a large meta-analysis (70), there were no differences in major or minor bleeding in patients treated or not treated with anticoagulants (11% for both groups), but a lower rate of variceal bleeding was observed in patients taking anticoagulants, maybe due to thrombus resolution in portal vein (70).

In patients with suspected variceal bleeding (37, 54), the use of vasoactive agents such as terlipressin, octreotide, or somatostatin at hospital admission is recommended and continued for a duration of up to 5 days. Furthermore, antibiotic prophylaxis is also recommended in patients with UGIB by suspected esophageal varices (37, 54). In particular, the European Society of Gastrointestinal Endoscopy (ESGE) suggests the use of ceftriaxone 1 g/day for up to 7 days for all patients with suspected variceal bleeding (or in accordance with local antibiotic resistance and patient allergies) (37). In addition, all patients should be stratified according to CHILD-PUGH and MELD scores and the endoscopic evaluation should take place within 12 h from the time of patient presentation/fluid resuscitation.

#### Endoscopic phase

Predictive pre- and post-endoscopic scores were developed during several years. In particular, pre-endoscopic score may help the physician to choose the optimal management of patients with UGIB evaluating an outpatient approach and estimating the risk of complications and death. In Table 2, we summarize the items of pre-endoscopic risk assessment scores recommended and validated in clinical practice (pre-endoscopic Rockall score, AIMS-65, and Glasgow Blatchford score) (54, 71–73). Recently, a simple ABC score (74) was proposed (Table 2), but not sufficient data are available to recommend it in clinical practice (54).

**Table 2:** Pre-endoscopic risk score for upper gastrointestinal bleeding.

Pre-endoscopic Rockall score (71)	AIMS-65 (72)	Glasgow Blatchford (73)	ABC score (74)
*Age (years) points* <60 + 0 60–79 + 1 ≥80 + 2	*Age (years) points* ≥65 + 1	*Blood Urea points* *(mmol/L)* ≥6.5 < 8.0 + 2 ≥8.0 < 10.0 + 3 ≥10.0 < 25.0 + 4 ≥25 + 6	*Age (years) points* 60–74 + 1 ≥75 + 2
*Shock signs* *(SBP mmHg, HR bpm)* *No shock* SBP ≥100 + 0 HR < 100 *Tachycardia* SBP ≥ 100 + 1 HR ≥ 100 *Hypotension* SBP < 100 + 2	*SBP (mmHg)* ≤90 + 1	*SBP (mmHg)* 100–109 + 1 90–99 + 2 <90 + 3	*Blood tests* Urea >10 mmol/L + 1 Albumin 150 μmol/L + 2 Creatinine 100–150 μmol/L + 1 >150 μmol/L + 2
*Comorbidities* None +0 - HF/ischemic +2 Heart disease/ Any major comorbidity Kidney/liver failure +3 Disseminated cancer	*INR* >1.5 + 1	Hemoglobin (g/L) for men ≥120 < 130 + 1 ≥100 < 120 + 3 <100 + 6	*Comorbidities* Altered mental status +2 Liver cirrhosis +2 Disseminated malignancy +4
Full Rockall score (71)	*Confusion* + 1	*Hemoglobin (g/L) for women* ≥100 < 120 + 1 <100 + 6	ASA score 3 + 1 ≥4 + 3
*Diagnosis* Mallory-Weiss tear +0 No lesion and no SRH All other diagnosis +1 Malignancy of UGI tract +2	*Albumin* <3 gr/dL +1	*Other markers* HR 100 bpm +1 Melena +1 Syncope +2 Hepatic disease +2 HF +2	
SRH None or dark spot only +0 - Blood in UGI tract +2 adherent clot Visible or spurting vessel			
Total score points Pre-endoscopic 7 Full 11	Total score points 5	Total score points 23	Total score points 14

HF, heart failure; HR, heart rate; SBP, systolic blood pressure; SRH, sign of recent hemorrhage; UGI, upper gastrointestinal.

In particular, a recent multicenter study involving 3,012 consecutive patients with UGIB showed that the Glasgow-Blatchford score has high accuracy at predicting need of hospitalization or death. Furthermore, a score of ≤1 is the optimum threshold for choose an outpatient management (75). For this reason, international guidelines recommend this score as first choice (37, 69, 76, 77).

In patients with UGIB candidate to endoscopy in emergency setting, this should be performed within 24 h of presentation (within 12 h if variceal bleeding is suspected) (37) and hemostatic endoscopic treatment is recommended only for ulcers with active spurting, active oozing, and non-bleeding visible vessels (37), while it is unclear whether endoscopic hemostatic treatment is useful for ulcers with adherent clot resistant to vigorous irrigation (37). No endoscopic treatment indicated whether only flat pigmented spots or clean base is found during endoscopy (69).

Finally, in patients with recurrent bleeding, after previous successfully endoscopic procedure, a new endoscopic treatment with clips is recommended, although with low quality of evidence (37).

### Post-endoscopic phase

In the post-endoscopic phase, medical therapy should be administered to reduce the risk of rebleeding and death. All guidelines recommend the use of high dose of PPIs (54) without the difference between continuous and intermittent regimen (37) (Figure 1).

The American College of Gastroenterology (ACG) guidelines recently suggest a medical therapy for UGIB based on endoscopic features (69). While the treatment of active ulcers or adherent clot findings is coherent with other guidelines (a high-intensity PPIs: for continuous regimen, 80 mg bolus followed by 8 mg/h infusion for 3 days and for intermittent regimens, 40 mg 2–4 times daily for 3 days, orally if feasible, after an initial bolus of 80 mg) (69), ACG guidelines for flat pigmented spot or clean base suggest standard dose-regimen PPI (69).

After high-dose PPIs, in patients undergoing hemostatic treatment, a further 2-week treatment with twice-daily PPIs is recommended to reduce rebleeding risk (37).

In addition, in case of proven variceal bleeding, vasoactive drugs should be continued for 2 to 5 days (54) (Figure 1).

In patients with variceal bleeding at high risk of recurrent bleeding following successful endoscopic hemostasis, pre-emptive transjugular intrahepatic portosystemic shunt (TIPS) within 72 h (preferably within 24 h) must be considered (37). NSBBs (propranolol or carvedilol) in combination with endoscopic therapy for secondary prophylaxis should be continued in patients with advanced chronic liver disease and/or and previous esophageal variceal bleeding.

## Management of acute lower GI bleeding

Lower gastrointestinal bleeding (LGIB) represents the 3% of emergency surgical referrals (78), and its incidence is estimated to be 33–87 for 100.000 patients (79). The mortality is 3.4% rising 18–20% in patients with LGIB during hospitalization (79). The most common cause of LGIB is diverticular bleeding, followed by benign anorectal conditions such as hemorrhoids, fissures, and rectal ulcers (79). Other common causes are telangiectasia in multiple sites of GI tract, colitis, and colorectal cancer. Of note, 23% of hospitalized patients with LGIB in the UK are discharged without a diagnosis (80). Patients with LGIB should be clinically evaluated to establish the hemodynamic stability. Clinical history (bleeding history and comorbidities), clinical evaluation (including digital-rectal exploration), laboratory test, and concomitant therapy are needed to establish the hemodynamic status of patients (81). In particular, shock index (heart rate [HR] and systolic blood pressure [SBP] ratio) is recommended by current guidelines (79). A shock index >1 defined the patient as hemodynamically unstable (79).

Similar to UGIB, a resuscitation strategy (previous described) should be performed in unstable patients (79). In these patients, a computed tomography (CT) scan with angiography should be performed to evaluate the focus of bleeding; then, patients should undergo to interventional radiology (preferably <60 min from hospital admission) or endoscopy (79). Although endoscopy treatment represents the first line of treatment from international guidelines (79, 81, 82), only 2.1% of cases of LGIB undergo endoscopic treatment and the most common intervention is red blood cell transfusions (79). If a treatment failure occurred during endoscopy or radiological intervention, surgery should be evaluated in selected cases. If no focus of bleeding is identified during CT scan with angiography, patients should be considered stable with major bleeding.

If shock index is <1, LGIB should be considered stable and Oakland score should be performed to establish whether major or minor bleeding occurred and whether hospitalization is required (Oakland score ≤ 8 suggests a possible outpatient management) (79).

**Figure 2:**
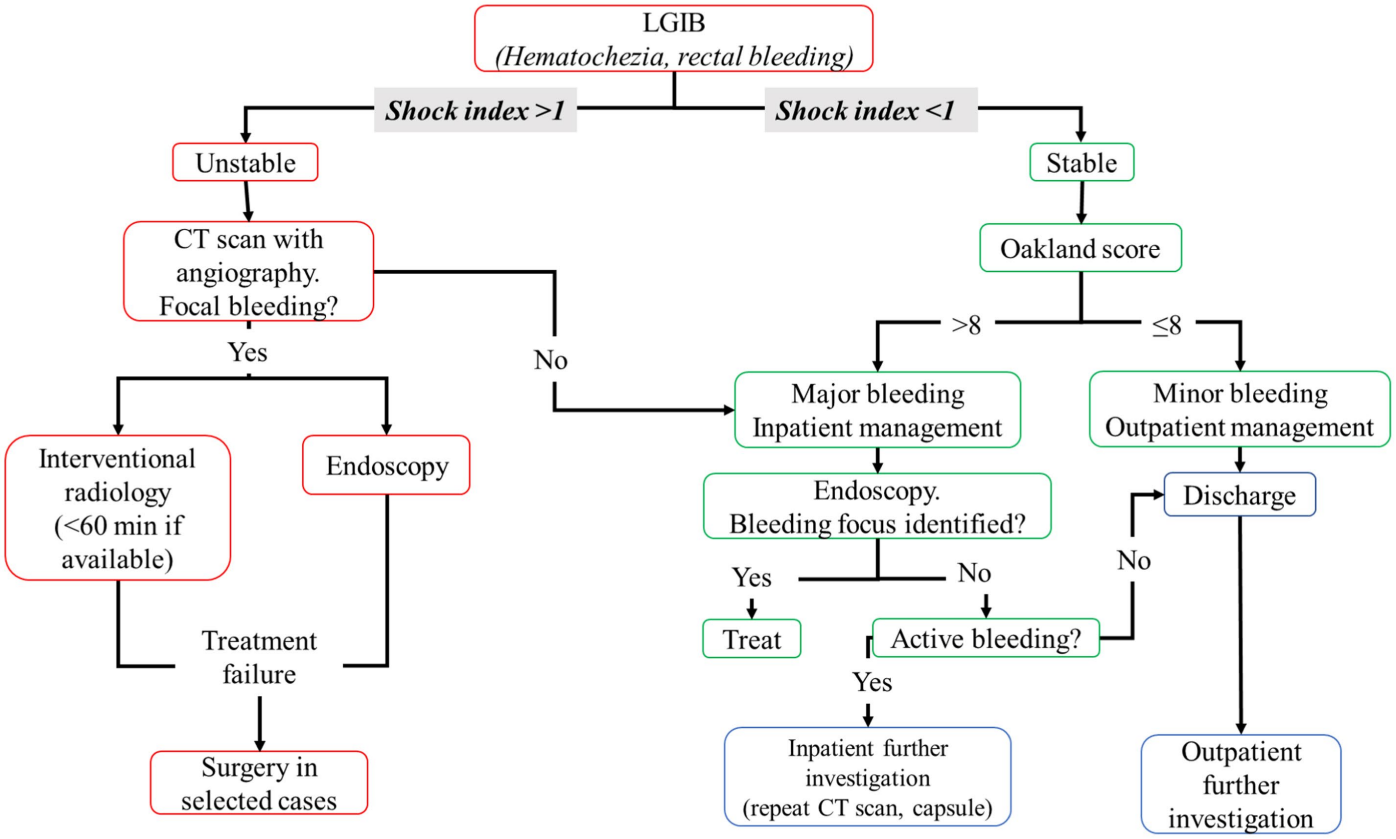
Flowchart of lower gastrointestinal bleeding management. CT, computed tomography; HR, heart rate; LGIB, lower gastrointestinal bleeding; SBP, systolic blood pressure.

Fluid resuscitation is needed, and a restricted red blood cell transfusion regimen should be preferred with a threshold of Hb <70 g/L with a target of 70–90 g/L after transfusion, except for patients with previous history of cardiovascular disease with a threshold of Hb <80 g/L with a target of 10.0 g/L (79), and platelets transfusion should be performed using a threshold of 50 × 10^9^/L (82). Figure 2 summarizes the management of LGIB.

In major bleeding, if patient is hemodynamically stable, colonoscopy should be performed after adequate colon cleansing (a nasogastric tube may help colon preparation in patients with a low risk of aspiration and ongoing bleeding) (82). In total, 4–6 liters of a polyethylene glycol (PEG)-based solution or the equivalent should be administered over 3–4 h until the rectal effluent is clear of blood and stool (82).

In patients with high-risk clinical features or ongoing bleeding, endoscopy should be performed within 24 h of patient’s admission to the emergency department, after an adequate colon preparation; otherwise, a colonoscopy should be performed next available after a colon purge (82). Further investigations are needed if no bleeding focus was found, such as CT scan with angiography repetition or use of video capsules (79).

## Management of GI bleeding in the older patients

In older people, the severity and prognosis of GI bleeding are influenced by medical comorbidities (1) and therapies as well as the use of antiplatelet and anticoagulants medication (83). Indeed, 70% of UGIB occurred in patients >60 years old and its incidence and mortality risk rise according to age (83). Similarly, patients with LGIB are more common in older patients, with a mean onset age between 63 and 77 years, with higher mortality risk (83). In addition, GI bleeding incidence seems to be reduced only in patients <70 years old (61). For these reasons, GI hemorrhage management is a backbone in the older care and there are some peculiarities of old age that should be addressed.

First, endoscopy, the first line of diagnosis and treatment for GI hemorrhage, has similar mortality risk in older patients compared to general population and old age is not a contraindication to endoscopy (83). Of note, older patients had an increased risk to developing adverse events and oxygen desaturation, especially if benzodiazepines (BDZ) are administered during endoscopy for sedation (83, 84); for this reason, a lower dose of BDZ with careful titration is suggested (83).

In addition, older patients are more likely to be treated with antiplatelets, anticoagulants, especially with complex antithrombotic therapy (CAT) resulting in an increased risk of hospitalization and transfusion as shown in a large cohort study of 78,133 old veterans aged >60 years treated with antiplatelets and/or anticoagulants, with the highest risk of hospitalization and transfusion in patients treated with dual antiplatelet agents and anticoagulant (85).

In patients treated with antiplatelets, a GI bleeding incidence rate of 0.7–1.3% for aspirin and 1.2–2% for aspirin and clopidogrel combination during a follow-up of 1–2 years was observed in Western countries (86–88). In older patients, the GI bleeding incidence rate rise to 2.7% as shown by an observational study on 1852 patients undergone the implantation of drug-eluted stent (DES) with a mean age of 70.9 years (89).

Antiplatelet management had a fundamental role in older patients. If antiplatelet is administered in primary prophylaxis should be permanently discontinued, while antiplatelet in secondary prevention should not be stopped, but if suspension is needed, it should be restarted when hemostasis is guaranteed (79, 82).

In patients treated with dual antiplatelet therapy with aspirin and a P_2_Y_12_ inhibitor, P_2_Y_12_ should be stopped only in unstable hemorrhage and restarted within 5 days, especially if recent coronary stenting is performed (54, 79, 81).

In older patients, comorbidities such as atrial fibrillation (AF) that require indefinite anticoagulation are common. The most common oral anticoagulant prescribed in older patients is vitamin K antagonists (VKAs) and direct oral anticoagulants (DOACs).

In older patients (≥75 years), the incidence rate of GIB was 2.19% per year for dabigatran 110 mg and 2.80% per year for dabigatran 150 mg (90). Instead, an incidence GIB rate of 1.51 and 0.83% per year was observed for edoxaban 60 mg and 30 mg, respectively (90). The incidence of GIB rate of 2.0% for rivaroxaban and 0.76% for apixaban, but no data according to dose were found (90). The incidence of GIB for warfarin ranged between 0.86 and 1.59% per year in phase III clinical trials (90). A large cohort meta-analysis performed on 129.357 patients by Miller et al. summarized the risk of GIB in patients treated with DOACs and VKAs (pooled rate: 1.5% versus 1.3%, respectively; odds ratio [OR]: 0.98; 95%CI: 0.80–1.21) (91).

Recently, a large network meta-analysis on 605.771 AF patients showed a reduced risk of GIB in patients treated with apixaban compared to ones treated with dabigatran or rivaroxaban (92).

VKAs, as warfarin, should be discontinued during hemorrhage and should be restarted at 7 days after GI bleeding if low thrombotic risk (54, 79, 81). In patients with high thrombotic risk as well as patients with mechanical prosthetic heart valve, AF with prosthetic heart valve or mitral stenosis, <3 months after venous thromboembolism, low molecular weight heparin treatment should be started at 48 h after hemorrhage (54, 79). In patients on warfarin, although the optimal international normalized ratio (INR) to perform endoscopy is <1.3, endoscopy may be also considered when INR is <2.5 without a significantly increased risk of rebleeding (54).

On the other hand, DOAC should be stopped at presentation of GI bleeding and restarted within 7 days after GI bleeding (54, 79, 81).

**Figure 3:**
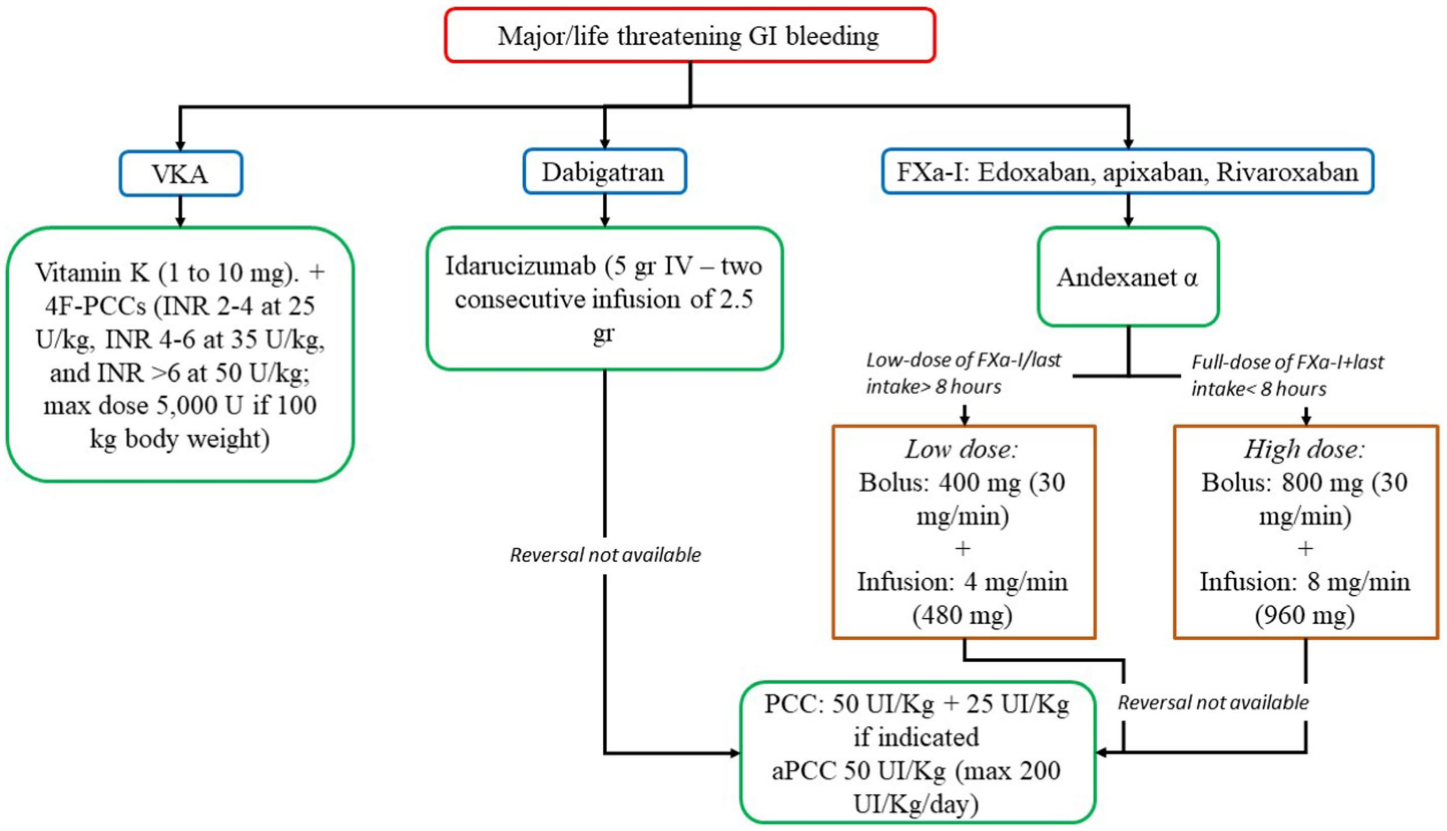
Reversal strategies for anticoagulants in major/life threatening gastrointestinal bleeding. 4F-PCC, 4-factor prothrombin complex concentrate; aPCC, activated prothrombin complex concentrate; FXa-I, inhibitors of FXa; GI, gastrointestinal; INR, international normalized ratio; PCC, prothrombin complex concentrate; VKA, vitamin K antagonist.

In life-threatening GI bleeding and hemodynamically unstable patients, the interruption of oral anticoagulants is not enough, and a reversal agent is needed (Figure 3) (12, 54, 79, 81).

In VKAs, Vitamin K is a specific reversal agent in a dose-dependent manner (1 to 10 mg). Slow intravenous administration (in 25 to 50 mL normal saline over 15 to 30 min) causes a rapid reduction in the INR (4–6 h) (93). However, the administration of vitamin K does not result in immediate correction of coagulopathy, and in life threatening bleeding, vitamin K administration must be accompanied by the administration of 4-factor prothrombin complex concentrate (4F-PCCs), or, if not available, plasma (93). 4F-PCCs should be administered according to INR range and body weight (INR 2–4 at 25 U/kg, INR 4–6 at 35 U/kg, and INR >6 at 50 U/kg; max dose 5,000 U if 100 kg body weight) (93).

In DOACs, in dabigatran users, a reversal agent, idarucizumab is suitable (5gr + 2.5gr, IV) (93). However, in patients taking apixaban, rivaroxaban, and edoxaban, andexanet alfa may be useful. If these drugs are not available, 4F-PCC or activated PCC (aPCC) may be an alternative (50 U/kg) (12, 93).

Furthermore, a large cohort study of 3,166 patients treated with antiplatelet and without routine PPI use, due to previous myocardial infarction or cerebrovascular event, showed that the long-term risk of bleeding is higher in older patients than in younger patients with a substantial risk of disabling or fatal UGIB, suggesting that a co-prescription of PPI should be encouraged (94).

On the other hand, the use of PPI is associated with several adverse effects, as well as increased risk of fractures, osteoporosis, higher risk of *Clostridium difficile* (CD) infection and community-acquired pneumonia (CAP), especially in older patients (95).

In particular, a large meta-analysis including 2,181,546 individuals taking or not taking PPI showed that patients not taking PPI, those taking PPI, had an increased risk of developing any-site fractures, hip fractures, spine fracture, and osteoporosis (96), this evidence is confirmed independently from dose and duration of therapy, as suggested by a large meta-analysis on 2,103,800 patients showing a high risk of hip fracture in patients with long- and short-term therapy and in low, medium, and high dosage of PPI (97).

In addition, PPI is also associated with an increased risk of developing CD infection as shown in a large meta-analysis of 56 studies (40 case control and 16 cohort) involving 356,683 patients (98). This risk is estimated approximately 64% compared to ones not taking PPI (99) and probably is related to PPI-gut dysbiosis (100, 101) that increases also all-cause mortality (101).

Finally, several studies showed an increased risk of developing CAP in patients taking PPI compared to ones who are not taking these drugs, particularly within 30 days (102–104). A pathogenic mechanism has been proposed to explain the association between PPI use and the incidence of CAP: PPIs may increase the gastric pH altering also normal oropharyngeal flora, which could increase susceptibility to respiratory infections by permitting survival of pathogens that lead to CAP (104).

Osteoporosis, bone fractures, CD infection, and CAP may be deadly for older patients, and these complications should be avoided. For this reason, PPI in older patients should be used only according to clinical indications, with long-term treatments only for selected cases (36).

## Conclusion

In conclusion, UGIB and LGIB represent a severe common complication, especially in older patients with comorbidities and on treatment with antiplatelet and/or anticoagulant drugs. Several drugs are available to reduce bleeding complications, especially for UGIB. Current evidence and guidelines suggest a clinical approach based on hemodynamic status with endoscopy as the first line for diagnosis and treatment.

## Author contributions

DM: Conceptualization, Writing – original draft. GG: Writing – original draft. FS: Writing – original draft. AP: Writing – review & editing. PP: Supervision, Writing – review & editing. DP: Conceptualization, Supervision, Visualization, Writing – review & editing.
